# Oral Health Literacy in Migrant and Ethnic Minority Populations: A Systematic Review

**DOI:** 10.1007/s10903-021-01266-9

**Published:** 2021-08-27

**Authors:** R. Valdez, K. Spinler, C. Kofahl, U. Seedorf, G. Heydecke, D. R. Reissmann, B. Lieske, D. Dingoyan, G. Aarabi

**Affiliations:** 1 Department of Prosthetic Dentistry, Center for Dental and Oral Medicine, University Medical Center Hamburg-Eppendorf, Hamburg, Germany; 2 Institute of Medical Sociology, Center for Psychosocial Medicine, University Medical Center Hamburg-Eppendorf, Hamburg, Germany; 3 Department of Periodontics, Preventive and Restorative Dentistry, Center for Dental and Oral Medicine, University Medical Center Hamburg-Eppendorf, Hamburg, Germany

**Keywords:** Oral health literacy, Dental service utilization, Oral health beliefs, Oral health behaviors, Oral health knowledge, Migrants, Ethnic minorities

## Abstract

Cultural background influences how migrants and ethnic minority populations view and assess health. Poor oral health literacy (OHL) may be a hindrance in achieving good oral health. This systematic review summarizes the current quantitative evidence regarding OHL of migrants and ethnic minority populations. The PubMed database was searched for original quantitative studies that explore OHL as a holistic multidimensional construct or at least one of its subdimensions in migrants and ethnic minority populations. 34 publications were selected. Only 2 studies specifically addressed OHL in migrant populations. Generally, participants without migration background had higher OHL than migrant and ethnic minority populations. The latter showed lower dental service utilization, negative oral health beliefs, negative oral health behavior, and low levels of oral health knowledge. Due to its potential influence on OHL, oral health promoting behavior, attitudes, capabilities, and beliefs as well as the cultural and ethnic background of persons should be considered in medical education and oral health prevention programs.

## Introduction

Due to the important interrelationship between oral and general health, oral health has been set as a Leading Health Indicator 2020 [1]. Oral inflammation (e.g. periodontitis) has been linked to non-communicable diseases such as cardiovascular diseases and diabetes [2–4], which both have a large impact on the health care economy [5]. The treatment of oral diseases can pose a great financial burden: not only at the individual level, but also for health care systems, as they are widespread and recurring [5]. In the European Union (EU) 79 billion EUR p. a. was spent on dental care between 2008 and 2012, which is expected to rise to 93 billion EUR in 2020 [5, 6]. Additionally, poor oral health has been shown to have a negative effect on quality of life [7–11].

Among other risk factors, having a migration background appears to be a risk factor for poor oral health [12–15]. Limited oral health literacy (OHL) is probably one reason for poor oral health in these populations. Current definitions of OHL have been based on the World Health Organization’s (WHO) definition of health literacy: “the cognitive and social skills which determine the motivation and ability of individuals to gain access to, understand and use information in ways which promote and maintain good health." [16]. Migrant populations usually represent a very heterogeneous group of persons with varying oral health knowledge, diverse beliefs and attitudes, shaped by their culture and past experiences with the respective health care system in their home countries. Therefore, these migrant populations may not fit well in the “health care culture” of their host country and subsequently do not sufficiently benefit from it. In fact, previous research has found that being a migrant had a profound effect on ones’ awareness of disease and health management. This awareness usually differs from the common health perceptions in the host country [17].

Various studies dealing with migrant or ethnic minority groups have reported beliefs and attitudes about oral health that may fundamentally shape the way they view and manage their oral health. For example, beliefs such as that retaining ones’ natural teeth during old age will bring misfortune to the family [18, 19] and that caries and tooth loss is part of a natural aging process [18] have been reported in Chinese immigrants in various host countries (e.g. Canada, England). A study investigating the oral health beliefs of Mexican-Americans regarding nutrition found that many staple foods with high amounts of sugar were not perceived to be rich in sugar (e.g. high carbohydrate foods, ketchup, sweet rolls) [20]. Thus, misconceptions and a resulting unhealthy diet may prevent persons from maintaining good oral health. Such differences in attitudes and beliefs may be a hindrance to interaction with the host country’s health care system and participation in health care interventions.

The influence of culture on OHL, as well as important components of OHL, can be explained by using the conceptual framework by Hongal et al. [21]. According to this framework, the management of one’s oral health, the patient-doctor interaction, oral health behaviors and attitudes, and the educational and health care system with which a person interacts – all affect one’s OHL. Furthermore, these factors additionally interact with one’s oral health knowledge, literacy, interests in oral health, and the ability to access oral health information and services. The societal, family, and peer influences within the different societies and cultures of migrants and ethnic minorities may positively or negatively affect their literacy skills in the language of the host country, thereby influencing their oral health knowledge, their ability to access oral health information and services, their oral health-related attitudes, and, subsequently, their OHL.

However, to date, only single studies have investigated OHL in migrant or ethnic minority populations and no review of this possible relationship and the specific determinants has been published. Therefore, this paper aims to systematically review and summarize research findings about OHL of adult migrant and ethnic minority populations in quantitative studies. The focus lays on adults, because previous research suggests that the OHL of caregivers (e.g. parents) plays an important role in ensuring good oral health in children [22–24]. Through targeted education of the parents, the oral health outcomes in children should be improved as well [24].

## Methods

The study was reviewed and approved by the ethics review committee at the Medical University Center Hamburg-Eppendorf (LPEK-0027). As the study does not involve human participants, human data, or human tissue, there were no ethical concerns.

The SPIDER (Sample, Phenomenon of Interest, Design, Evaluation, Research) method [25] was applied to generate the search strategy. The following terms related to the concept “migrant” were included (S): migrant*, migrat*, immigrant*, immigrat*, emigrat*, refugee*, ethnic*, and race. To assess the concept “oral health literacy” (PI), the terms “oral, dental, literacy, knowledge, coping, self-management, health promotion, and health prevention” were used (Table 1).

**Table 1:** Search terms

Search terms	
Databases/Sources used (Date)	(Search string) N = 1652
Pubmed (7/18/2019)	(migrant* OR migrat* OR immigrant* OR immigrat* OR emigrant* OR emigrat* OR refugee* OR ethnic* OR race) AND (((oral OR dental) AND literacy) OR ((oral OR dental) AND knowledge) OR ((oral OR dental) AND “coping*”) OR ((oral OR dental) AND “self-management”) OR ((oral OR dental) AND “health prevention”))

Because of the lack of research on this topic and the possibility of unintentionally excluding relevant studies, no restrictions were applied to the search strategy in terms of the evaluation (E) of the publications. Original quantitative publications were included (D, R). No time restriction was applied as an exclusion criterion. Only publications in German or English were included. During the initial title screening, all publications unrelated to oral health were removed. During abstract and full text screening, studies were excluded, which had no focus on migration/ethnicity/race, included persons under 18 years old, included data other than original quantitative data, did not deal with OHL or at least one of its indicators (e.g. oral health knowledge, dental service utilization, oral health beliefs), or focused only on clinical health status instead of OHL. A criteria list for the abstract and full text screening was developed and used by the two reviewers for the screening of abstracts and full texts. Any discrepancies found during the selection of the full texts to be included in the review were discussed and resolved.

## Results

**Fig. 1:**
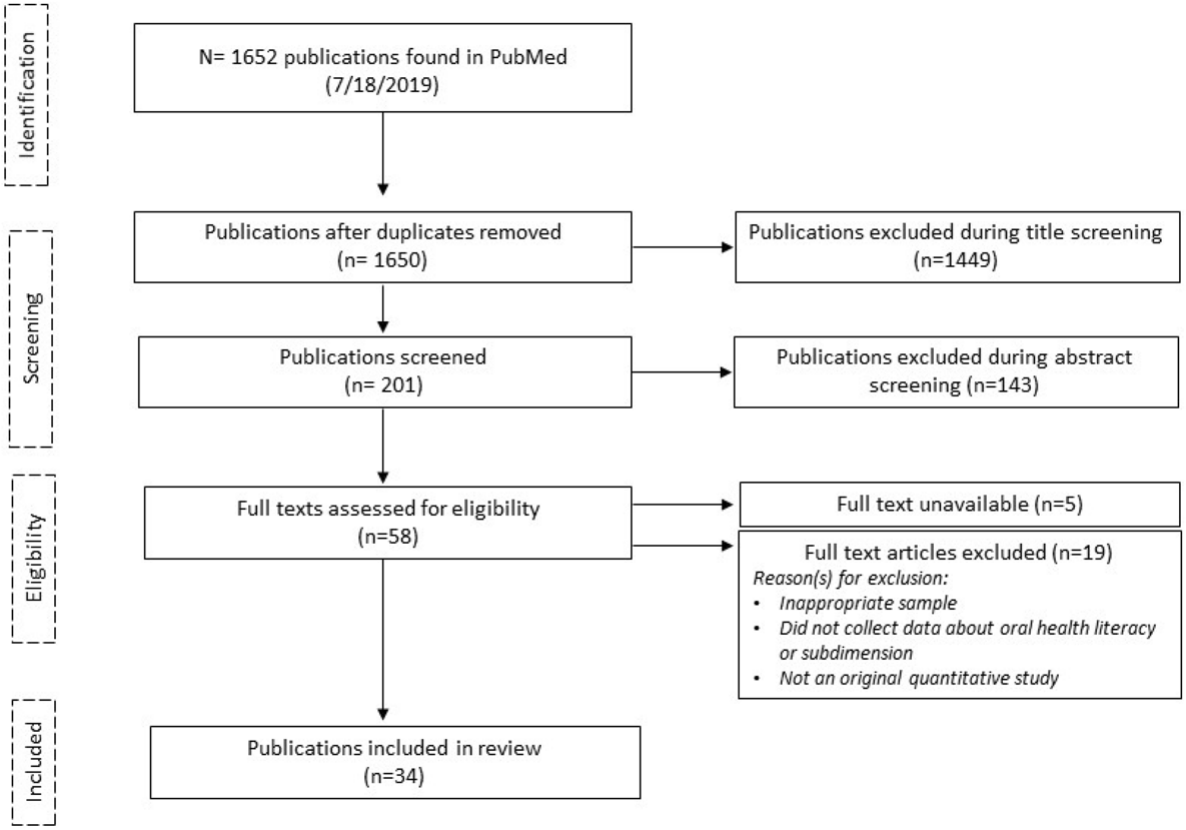
PRISMA flow diagram

A total of 201 publications was selected for abstract screening, resulting in 58 publications for the full text screening. Of these 58 publications, 5 full texts could not be found despite contacting the authors, and 19 were excluded from the review based on the criteria listed in Fig. 1. This review includes a total of 34 publications, originating from industrialized countries like Australia, Austria, Canada, China, Israel, Germany, Norway, Sweden, and the United States (US), with the majority coming from the US (N = 24).

### OHL Studies in Migrants and Racial/Ethnic Groups

Only 8 studies specifically explored OHL, originating from the US (N = 7) and Canada (N = 1). Of these 8 studies, only 2 studies specifically targeted *migrant* populations [26, 27], while all others collected data about *ethnicity* or *race* [28–33]. Measurement of OHL or HL in dentistry in these studies consisted mainly of word recognition tests (e.g. REALM-D [34] for dental-related terms or S-TOFHLA, a generic test of functional literacy in adults [35]).

Geltman and colleagues [27] used the REALD-30 as a measure for HL in dentistry as well as the S-TOFHLA in a sample of Somali refugees, where 73% had low REALD-30 scores and 74% had low S-TOFHLA scores (Table 2). People with higher REALD-30 scores and higher English proficiency were twice as likely to visit the dentist for preventive purposes within the preceding year. However, these associations disappeared when controlling for the effects of acculturation and stratifying by sojourn time in the US.

**Table 2:** Oral health literacy studies in migrants and racial/ethnic groups

	Author Year	(Host) country	Study design; sampling	Sample (N) Refs. (N)	(O)HL instrument	Analyzed factors	Main results
1	Atschinson et al. 2010	USA	Instrument development; convenience sampling	Asian/Pacific Islanders, Hispanic, and AA dental patients (N = 200); Refs.: Caucasians (N = 115)	REALM-D	Health beliefs, health attitudes, ethnicity, education, main language	OHL: (+) education, (+) English competency REALM-D score differences: non-Caucasian participants < Caucasian participants
2	Burgette et al. 2016	USA	Cross sectional; purposeful sampling of diverse backgrounds	Female AA, American Indian/Alaskan, Asian caregivers (N = 1,277) Refs.: Caucasians (N = 499)	REALD-30	Race, marital status, self-efficacy, education, age	OHL: (+) education, (+) self-efficacy, (+) race, (-) dental service utilization (-) marital status, (-) age REALD-30 score differences: non-Caucasian participants < Caucasian participants
3	Calvasina et al. 2016	Canada	Cross sectional; snowball sampling	Brazilian immigrants (N = 101) No refs	OHLI	Age, gender, education level, Canadian education (Y/N), job status, income, length of stay, OH self-report, OH information sources, OH efficacy, HL, DSU, dental info seeking behavior, dental treatment decision making	OHL: (+) not visiting dentist, (+) not having dentists as source of information, (+) participation in dental treatment decision making Mean OHLI score: 85.5 (“adequate OHL”)
4	Divaris et al. 2011	USA	Cross sectional; quota sampling	Female AA, American Indian caregivers (N = 1,405) Refs.: Caucasians (N = 503)	REALD-30	OHrQoL, self-efficacy, age, education level, race	OHL: (+) education, (+) race, (+) OH-related quality of life REALD-30 score differences: non-Caucasian participants < Caucasian participants
5	Gelten et al. 2014	USA	Cross sectional; convenience sampling	Somali refugees (N = 439) No refs	S-TOFHLA REALD-30	Gender age, ethnicity, education level, income, dental insurance (yes/no), OHrQoL	HL: (+) preventive dental visits in last year OHL: (+) preventive dental visits in last year, (+) English competency Low REALD-30: 75% Inadequate S-TOFHLA: 74%
6	Jackson et al. 2008	USA	Cross sectional; convenience sampling	AA dental study volunteers (N = 98) Refs.: Caucasians (N = 58)	S-TOFHLA	Race, gender	HL: (+) age, (+) gender, (+) race S-TOFHLA score differences: female Caucasians > male AAs
7	Messadi et al. 2018	USA	Cross sectional; convenience sampling	Hispanic, AA, Asian, “Other/Mixed Race” dental patients (N = 793) Refs.: Caucasians (REALM-D N = 310; S-TOFHLA N = 298)	S-TOFHLA REALM-D	Age, gender, education, race/ethnicity, income, dental insurance, speak English as a child, marital status, preventive behavior, health services index, smoking, locus of control, info seeking behavior, medical history	OHL: (+) education, (+) English competency REALM-D score differences: non-Caucasian participants < Caucasian participants S-TOFHLA score differences: Asians > Caucasians > AAs > Hispanics
8	Tam et al. 2015	USA	Cross sectional; convenience sampling	AA, Asian, Hispanic dental patients (N = 100) Refs.: Caucasian (N = 42)	REALMD-20 REALM-D	Gender, age group, race, education level, OH knowledge	OHL: (+) education, (+) race/ethnicity, (+) OH knowledge, (-) age, (-) gender Mean REALM-D: 23 (out of 28) Mean REALMD-20: 17

OH = oral health, OHL = oral health literacy, HL = health literacy, DSU = Dental service utilization, OHrQoL = oral health-related quality of life, AA = African Americans

[ +] association found; [–] no association found

Calvasina [26] reported that 83.1% of Brazilian immigrants living in Canada who participated in their study had adequate OHL as measured by the OHLI developed by Sabbahi et al. [36], which contains numeracy and reading comprehension items. However, 46.5% of the participants had inadequate oral health knowledge. Limited OHL was associated with not visiting a dentist in the preceding year, not having a dentist as a primary information source, and not participating in shared dental treatment decision making. English comprehension in this sample is implied to be low. The majority (86.1%) of participants in this study chose to complete the questionnaire in Portuguese (Tables 3 and 4).

**Table 3:** Studies investigating dental service utilization, oral health behavior, oral health beliefs, and oral health knowledge in migrants

	Author Year	Host country	Study design; sampling	Sample (N) Refs. (N)	OHL indicator(s)	Analyzed factors	Main results
1	Cruz et al. 2010	USA	Cross sectional; non-probability snowball sampling	Asian & Hispanic immigrants (N = 1,417) No refs	DSU, OH Behavior	Sociodemographic data, self-perceived OH, immigration status, clinical OH outcomes	DSU: > 70% in all groups had no regular source of dental care, no dental insurance, > 75% did not visit the dentist within the last 12 months DSU: (-) age at immigration, (-) gender, (+) flossing, (+) dental insurance & (+) having a regular source of dental care (AAs Caribbean only), (+) more filled teeth OH Behavior: > 40% do not floss (all ethnic groups)
2	Gao et al. 2014	China	Cross sectional; cluster random sampling	Indonesian domestic helpers in Hong Kong (N = 122) No refs	DSU, OH Behavior, OH Beliefs	Gender, age, education level, fluency in Cantonese and/or Mandarin, immigration history (residence in other Chinese society, yes/no), living condition (Having one's own room in employer's home, yes/no), family in Hong Kong (yes/no), leisure activities (shopping/religious gatherings, shopping/exercise/rest), clinical OH outcomes, OH self-efficacy	DSU: 93% reported going to the dentist irregularly (problem oriented) OH Behavior: 81% snacked or had meals 3 times a day or less, 97% toothbrush 2 × daily, 77% never floss OH Beliefs: 100% believe OH is important, 96% believe regular checkups prevent dental problems, 64% believe tooth loss a natural aging process
3	Kohlenberger et al. 2019	Austria	Cross sectional; random sampling	Syrian, Iraqi, Afghan refugees (N = 515) Refs.: Austrian residents (N = 11,425)	DSU	Namely self-reported health, access to health services, satisfaction with health services, psychosocial stress and resulting restrictions, discrimination experiences, and demography	DSU: 27% male and 28% female refugees reported not consulting a dentist within the last 12 months
4	Lai et al. 2007	Canada	Cross sectional; random sampling of telephone numbers listed with Chinese surnames	Older Chinese immigrants (N = 1,537) No refs	DSU	Age, post-secondary education, length of residency in Canada, country of origin, social support, lived in Quebec, self-reported physical/mental health, self-reported dental problems	DSU: < 59% used dental services DSU: (+) high education, (+) host country language competency, (+) length of residency, (+) high social support, (+) better physical and mental health, (+) existing dental problem, (-) residency in Quebec, (-) immigration from Taiwan
5	Marino et al. 2005	Australia	Cross sectional; convenience sampling	Greek & Italian immigrant (N = 734) No refs	DSU, OH Knowledge, OH Beliefs	Age, gender, level of education, occupation before retirement, living, clinical OH outcomes, physical health score, mental health score, OH treatment need score, attitudes to health care score	DSU: 40% Greek and 45% Italians visited the dentist within the last 12 months. 98% did not visit the dentist in the last two years DSU: (+) higher # of teeth, (+) host country language fluency, (-) dental cost, (-) perceived difficulty getting a dentist appointment OH Knowledge: Mean scores (out of 36) = 11.6 (Greeks), 12.9 (Italians) OH Beliefs: Mean scores (higher values represent more positive OH beliefs) = 3.0 (Greeks), 2.9 (Italians)
6	Nguyen et al. (2017)	USA	Cross sectional; convenience sampling	Vietnamese immigrants (N = 140) No refs	OH Beliefs, OH Behaviors, DSU	Age, gender, primary language, years spent in the United States, education level, religion	DSU: 67% visited the dentist within the last 2 years; 53% had dental cleaning within the last 2 years OH Beliefs: > 80% believed that regular dental visits prevent dental problems; 90% believed keeping natural teeth is important; > 80% believed losing teeth/bleeding gums are serious matters; 75% believed that total tooth loss is a natural aging process; 77% believed excess consumption of hot food causes dental problems OH Belief “Losing teeth is a serious matter”: (+) last dental visit; (+) last physical OH Behaviors: 88% would visit the dentist due to gum bleeding; 75% would not visit the dentist because of toothache; 89% use home remedies for oral health problems, 66% had family that used home remedies for oral health problems
7	Okunseri et al. 2007	USA	Cross sectional; location sampling	Hmong refugees (N = 118) No refs	DSU, OH Behavior	Age, gender, marital status, education level, income per year, dental insurance status, language preference, foreign born (yes/no), duration of stay in US	DSU: 43% visited the dentist within the last 12 months, 25% within the last 2–3 years, 47% went for regular checkup, 39% had no regular source dental care, 86% would visit the dentist instead of a traditional healer OH Behavior: 80% tooth brushed 2 × daily
8	Selikowitz et al. 1986	Norway	Cross sectional; convenience sampling	Pakistani immigrants (N = 160) No refs	DSU, OH Beliefs, OH Knowledge	Perceived dental health, age group, place of origin (city, town, or village), gender, number of years in Norway, income, clinical OH outcome	DSU: 60% visited the dentist within the last 3 years, 54% went to dentist due to pain DSU: (+) no perceived dental problem, (+) longer duration of stay, (+) dental cost, (+) perceived difficulty getting a dentist appointment OH Beliefs: 83% do not believe dental disease to be dangerous OH Knowledge: 64% correctly answered question about etiology of dental disease
9	Solyman et al. 2018	Germany	Cross sectional, location sampling	Refugees from Syria and Iraq (N = 386) No refs	OH Beliefs, OH Behavior	Country of origin, gender, age group, education level, OH status outcomes	OH Behavior: 59% tooth brush 2 × daily, 98% use a toothbrush instead of miswak OH Beliefs: 91% believed tooth brushing improves dental health, 69% believed one should not floss in addition to brushing, 54% believed one should only go to dentist if there is a problem
10	Vered et al. 2008	Israel	Longitudinal-cohort (1999–2000 and five years later from 2004–2005), no sampling method described	Ethiopian immigrants (N = 792) No refs	OH Behavior	Clinical OH outcomes, age group, gender	OH Behavior: At baseline, 74% reported cleaning their teeth exclusively utilizing chewing and cleaning sticks common in Ethiopia. After five years, 97% reported cleaning their teeth exclusively utilizing toothbrushes
11	Widstrom et al. 1984	Sweden	Cross sectional; random sampling	Finnish immigrants (N = 1,002) No refs	DSU	Age group, gender, years in Sweden, social class	DSU: 73% women 78% men visited the dentist, 45% men and 55% women had a dentist, 5.5% regularly went to the dentist (within 1–2 years) DSU: (+) longer duration of stay, (+) fluency in host country language, (-) unfamiliarity of dental health system, (-) perceived difficulty getting a dentist appointment
12	Wilis et al. 2011	USA	Descriptive questionnaire research; convenience sample from existing contacts during questionnaire development	Refugees from Sudan (N = 32) No refs	DSU, OH Behavior	Education level, dental coverage type, ethnic group, length of residency in the US, diet, dental aesthetics	DSU: 56% visited a dental facility only once since arriving in host country, 0% report visiting dentist for biannual check-up OH Behavior: 44% reported tooth brushing 1 × daily, 18% 2 × daily, 21% 3 × daily, 80% reported using traditional oral hygiene tool (toothbrush tree) 2 × daily before coming to host country
13	Wu et al. 2005	USA	Cross sectional; convenience sampling	Older Chinese and Russian immigrants (N = 477) No refs	DSU	Insurance coverage, physical and mental health, social support, risk behaviors, age, gender, living arrangements, education level, income, length of stay in the US, English competency	DSU: Fewer Chinese immigrants used dental services within the past year than Russian immigrants DSU (Chinese): (+) education, (+) duration in US, (+) social support, (-) smoking DSU (Russian): (+) age, (+) income, (+) denture use
14	Xhihani et al. 2017	USA	Cross sectional, purposive sampling	Albanian immigrants (N = 211) No refs	DSU, OH Beliefs	Age, gender, first language, predominant language, country of birth, years living in USA, marital status, education level, country where educated, dental insurance, OH beliefs, use of preventive services and home remedies	DSU: 68% visited the dentist within the past year, 89% possess dental insurance OH Beliefs: > 50% did not believe tooth loss a natural aging process, > 80% believed it is important to retain natural teeth, that tooth loss and bleeding gums are serious matters, and > 90% believed regular dental visits prevent dental problems

OH = oral health, OHL = oral health literacy, HL = health literacy, DSU = Dental service utilization, OHrQoL = oral health-related quality of life

[ +] association found; [–] no association found

**Table 4:** Studies investigating dental service utilization, oral health behavior, oral health beliefs, and oral health knowledge in ethnic minority groups

	Author Year	Country	Study design; sampling	Sample, ethnic groups (N)	OHL indicator(s)	Analyzed factors	Main results
1	Atschinson et al. (1997)	USA	Cross-section; probability sampling	AA, Caucasian, Hispanic, non-Hispanic, American Indian (N = 2,291) Refs.: Caucasian (N = 814)	OH Beliefs	Sociodemographic characteristics: Ethnicity, age, gender, years of education, marital status; enabling resources: household income, dental insurance (Y/N), usual source of dental care; perceived need for dental treatment: dentures, teeth/gum pain (Y/N), # of oral symptoms; Predisposing oral health beliefs: Perceived seriousness of disease, benefit of preventive practices, efficacy of dental, perceived importance of oral health, not afraid of dental pain, will go to dentist even if busy (motivation), dentists are always available	OH Beliefs: Caucasians believe that oral disease more seriously than Hispanic adults, and had significantly stronger beliefs about preventive practices than most ethnic minority groups, ex. benefit of plaque control Hispanics were less likely to believe that oral health is important in comparison to Caucasian adults. Older Hispanics were significantly less likely to believe in the benefit of plaque control than Caucasian adults
2	Boggess et al. (2010)	USA	Instrument development; convenience sampling	Pregnant women; Caucasian, AA, Hispanic, Other, More than one race/ethnicity (N = 599) Refs.: Caucasian (N = 248)	DSU, OH Behavior	Age, trimester, race/ethnicity, education level, annual household income, insurance coverage	DSU: 25% Hispanic and 16% AA women never received dental care vs. only 5% Caucasian never received dental care; Hispanic least likely to receive routine dental care during pregnancy DSU: (+) Hispanic ethnicity, (+) income, (+) education OH Behavior: AA more likely than Caucasians and Hispanics to tooth brush teeth less than 1 × daily;
3	Boggess et al. (2011)	USA	Instrument development; convenience sampling	Pregnant women; Caucasian, Hispanics, AA, Asian and 'other race' (N = 599) Refs.: Caucasians (N = 253)	OH Knowledge, OH Beliefs	Age, trimester, race/ethnicity, education level, annual household income, insurance coverage, country of origin, marital status	OH Knowledge: Hispanic women had significantly lower knowledge scores than Caucasian and AA women OH Beliefs: Mexico-born women had significantly lower beliefs scores than women born in the USA
4	Brega et al. (2019)	USA	Cluster randomized trial; random sampling	American Indians and Alaska Natives (N = 990) No refs	OH Knowledge, OH Beliefs	Parental ethnic identity; parents’ oral health knowledge, attitudes, and behavior; oral health outcomes; and sociodemographic characteristics	OH Knowledge: 74% of correct answers on average OH Beliefs: Agreed about the importance in engaging in good oral health behaviors (mean answer = 4.7), poor health is a severe problem (mean answer = 4.3), perceived benefits in good oral health behavior (mean answer = 4.3)
5	Davidson et al. (1997)	USA	Cross sectional; quota sampling	Hispanics, AAs, American Indians (N = 2729) Refs.: Caucasians (N = 1675)	DSU, OH Beliefs	Race/ethnicity, age cohort, gender, education level, marital status, general health, dentate, edentulous, income, usual source of dental care, presence of oral pain or symptoms	DSU: Only 42% American Indian (Navajo), 52% American Indian (Lakota), 42% Hispanics, and 57% AA visited the dentist in the last 12 months, while 80% of Caucasians did (dentate adults between 35–44 years) DSU: Only 29% American Indian (Navajo), 36% American Indian (Lakota), 37% Hispanics, and 48% AA visited the dentist in the last 12 months, while > 70% Caucasians did (dentate adults between 65–74 years) Predictors of DSU: (+) No fear/pain, (+) education (Caucasian only), (+) dentate status, (+) motivation to visit dentist even if busy, (+) usual source of dental care, (+) oral pain symptom
6	Gilbert et al. (1997)	USA	Longitudinal; purposeful sampling	N = 873 AA: 28% Caucasian: 72%	DSU, OH Knowledge, OH Beliefs	Gender, age, residency in a rural or urban area	DSU “Poor”: 40% AA reported last dental visit to be 5 + years ago vs. 29% Caucasians; only 11% AA and 21% Caucasians went for a yearly dental checkup in the last 5 years DSU “Not Poor”: 30% AA reported last dental visit to be 5 + years ago vs. 9% Caucasians; only 21% AA and 58% Caucasians went for a yearly dental checkup in the last 5 years OH Knowledge: Only 22% poor AAs and 49% not poor AAs report knowing what a root canal is vs. 55% poor Caucasians and 83% not poor Caucasians OH Beliefs: AA and Caucasians believe in the importance of dental visits, effectiveness of dental care, the eventuality of dental decline, and personal influence of dental decline
7	Junger et al. (2019)	USA	Longitudinal; random sampling	Caucasian, AA, Hispanic, other (N = 3,550) Refs.: Caucasian (66.3%)	OH Knowledge	1. General media habits, product use, interests and lifestyle 2. Health orientations and practice 3. The extra question “Which of the following best describes the purpose of dental sealants?” All weighted by sex, age, household size, education, census region, metro status and prior internet access	OH Knowledge: 66% Caucasians, 11% AA, 15% Hispanic, 8% “Other” were aware of the purpose of dental sealants OH Knowledge: (+) race, (+) income, (+) education
8	Kiyak et al. (1981)	USA	Cross sectional; convenience sampling	Caucasians, Chinese, Vietnamese, Thai, Lothian Korean living in the US (N = 96) Refs.: Caucasian (N = 46)	OH Knowledge, OH Beliefs, OH Behavior	Oral health status, ethnicity, education level, marital status, age	OH Knowledge:72% Asians denoted poor oral hygiene as the cause of caries while 64% Caucasians denoted it as a decay process; < 50% Asians knew the etiology of periodontal disease and tooth loss while > 70% Caucasians did OH Behavior: 76% Asians flosses 2 × daily vs. 49% Caucasians; 56% Asians vs. 82% Caucasians never floss; 26% Asians floss 2 × daily vs. 9% Caucasians; 10% Asians never consume cariogenic foods vs. 48% Caucasians
9	Lee et al. (1992)	USA	Cross sectional; purposeful sampling	Korean-American (N = 43) No refs	DSU, OH Beliefs	Age, education, level, length of time in USA, perceived self-efficacy in performing dental health behaviors, oral health status (reported by dentist), dental attitudes, definition of disease processes, preventive health orientation, self-reported dental practices,	DSU: Mean months since last dentist visit in Korean-American between 20–45 years = 31 months vs. 8.9 months for Koreans aged 60 + ; Mean months since last preventive dental checkup in younger Korean-Americans = 39 months vs. 54 months in older Koreans OH Beliefs: Both age groups showed positive OH beliefs about preventive practices (score > 70%) and that dental health is most important (mean score > 4 out of 5)
10	Macek et al. (2017)	USA	Cross-sectional, convenience sampling via dentist’ files	"Caucasian, AA, Asian, “Non-Hispanic Other,” Hispanic (N = 909) Refs.: Caucasians (N = 347)	OH Knowledge	Recruitment site, age group, gender, race/ethnicity, education level, language(s) spoken as a child, self-efficacy to prevent caries and periodontal disease	OH Knowledge: 68% Caucasians, 48% AAs, 49% Asian, 51% Non-Hispanic Other, 44% Hispanics had Middle-Low to Middle High OH knowledge
11	Payne et al. (1994)	Canada	Cross-sectional; random sampling via voting lists	Canadian, British, Italian, Jewish, Caribbean, Chinese, AA, Hispanic, East Indian or Vietnamese, other (N = 1,050) Refs.: Canadians (18.8%)	DSU, OH Behavior	Age group, gender, place of birth (Canada or Outside of Canada), mother tongue, ethic/racial origin, married, live alone, income level, dentate/edentulous, education level, oral problems (pain, chewing), perceived need for dental care, dental insurance coverage	DSU: > 70% of Canadians, British, Jewish, Other had a preventive dental checkup within the past 12 months vs. 39% Italians; OH Behavior: > 60% of Canadians, British, Jewish, Other tooth brush 2 × daily vs. 38% Italians; > 20% of Canadians, British, Jewish, Other floss 2 × daily vs. 15% Italians
12	Shelley et al. (2011)	USA	Cross-sectional; random sampling	Caucasian, AA, Hispanic, Asian (Chinese) (N = 1,722) Refs.: Caucasians (N = 725)	DSU	Gender, age, education level, born in US, years since immigration, English spoken at home, ability to speak English, insurance status,	DSU: Caucasian higher dental care utilization compared to all other racial/ethnic groups DSU: (+) Host country language competency

OH = oral health, OHL = oral health literacy, HL = health literacy, DSU = Dental service utilization, OHrQoL = oral health-related quality of life, AA = African Americans

[ +] association found; [–] no association found

OHL-studies collecting only race/ethnicity data found that high education and English competency were associated with higher scores in REALM-D [28, 32] and REALD-30 [30] in non-Caucasian participants than in Caucasian participants. For instance, one study observed significantly higher REALM-D scores in non-Hispanic Caucasians than Hispanics [32]. The study by Tam et al. [33] also observed significant associations between OHL (REALMD-20 & REALMD) and race/ethnicity as well as OHL and education. Another study using the S-TOFHLA within a dental research context [31] observed that Caucasian females had higher HL scores than African American males. Moreover, higher age was also associated with lower HL. Messadi et al. [32] also collected ethnicity/race data and observed high S-TOFHLA (S-TOFHLA score > 22) mean scores in all ethnic groups. However, the scores were highest in non-Hispanic Asians, followed by non-Hispanic Caucasians, African Americans, and Hispanics. In a sample of ethnically diverse female caregivers, no significant associations between OHL (REALD-30) and dental service utilization were detected [29].

### Studies Investigating Dental Service Utilization, Oral Health Behavior, Oral Health Beliefs, and Oral Health Knowledge in Migrants

The majority of studies investigating at least one component of OHL in migrant populations collected data on dental service utilization [14, 37–47]. Six of these studies took place in the US; they show different results in various migrant populations. A study by Xhihani et al. [46] that explored the dental service utilization of Albanian immigrants (mean duration of stay in US = 12.9 years) observed high utilization of dental services, with 68% of this group having visited the dentist within the past year. Wu et al. [47] investigated the dental service utilization patterns of older Chinese and Russian immigrants (60 + years old) in the US and found that both had a low service utilization rate. Among them, fewer Chinese elders (46.9%) had used dental services in the last 12 months than Russian elders (60.3%). Predictors were different in these groups. Education, length of stay in the US, social support, and smoking behavior were significant indicators for the use of dental services among older Chinese, while age, income, and denture use were significant indicators for dental service utilization in older Russian immigrants.

Another study in 2010 examining the determinants of oral health care utilization among a diverse group of immigrants in New York City observed that the majority of Asian, Hispanic, and African American Caribbean immigrants reported not having a regular source of dental care, not having dental insurance, and not having visited the dentist within the last 12 months (> 70% in all groups) [37]. A positive association between having a regular source of dental care and dental service utilization was observed in all ethnic groups.

Other US-studies focused on various refugee populations. In 2007, Okunseri and colleagues reported that 39% of Hmong refugees did not have a regular source of dental care and only 43% had visited the dentist within the last 12 months [42]. A study involving refugees from Sudan [45] reported that 56% of participants had used dental services only once since arriving in the US (the duration in the US ranged between 10–13 years). None of them reported going to the dentist for a biannual checkup [45].

Further studies outside of the US were focusing on: Chinese immigrants in Canada [39], Indonesian workers in Hong Kong [48], Greek and Italian immigrants in Australia [40], Pakistani immigrants in Norway [14], Finish immigrants in Sweden [44], refugees from Syria, Iraq, and Afghanistan in Austria [38]. All these studies revealed a low dental service utilization among migrants. However, the predictors for dental service utilization varied between these migrant populations. Level of education [39], number or condition of remaining teeth [14, 40], duration of stay in the host country [14], fluency in the host country’s language [38–40, 44, 48], costs of dental services [14, 40], familiarity with the host country’s dental health care system [44], and possibilities in getting a dental appointment [14, 40, 44] were reported as factors for (non-)utilization of dental services.

A few studies also observed oral health beliefs. In a study in Hong Kong, Indonesian workers reported to believe in the importance of regular dental check-ups [48], while the older Albanian immigrants in a study of Xhihani and colleagues [46] in the US did not believe retaining one’s teeth to be important and considered bleeding gums as normal. In Germany, the majority of Syrian and Iraqi refugees believed that oral diseases can affect general health and, thus, tooth brushing improves health [49].

Several studies collecting data on oral health behavior in migrants reported that flossing the teeth is rare to non-existent [37, 48], while regular tooth brushing (twice a day) seems to be quite common [42, 48, 49]. Nevertheless, despite brushing the majority of participants in the two studies that assessed oral hygiene had plaque/calculus [48, 49]. Due to the findings of Gao et al. as well as of Vered et al. the oral health behavior of immigrants can improve, such as more frequently flossing [48] or switching from traditional means of oral hygiene (e.g. chewing sticks) to toothbrushes [43].

Two studies measuring oral health knowledge found low scores in Greek and Italian migrants [40], while in another study in Norway more than half of a population of Pakistani immigrants were knowledgeable of questions about etiology of dental diseases [14].

### Studies Investigating Dental Service Utilization, Oral Health Behavior, Oral Health Beliefs, and Oral Health Knowledge in Racial/Ethnic Minority Groups

Studies investigating the dental service utilization in minority racial/ethnic groups in the US and in Canada (e.g. Hispanics, African Americans, Native Americans, Chinese-Americans) reported that these populations were less likely than Caucasians to obtain dental care [50–54]. Davidson et al. [51] reported different predictors of dental service utilization, such as fear, pain, and education, between ethnic groups.

Varying levels of oral health knowledge were observed in studies collecting data only about race/ethnicity. The ones performed in the US found that Caucasians typically had a better oral health knowledge than other racial/ethnic groups [52, 55–58]. On the other hand, high oral health knowledge was reported in samples of American Indians and Alaskan natives [59] as well as Korean-Americans [60].

Oral health beliefs also varied between different race/ethnic minority groups. Although most studies observed that ethnic/racial minority groups have negative oral health beliefs (e.g. not believing in the benefits of preventive dental care) [52, 55, 57, 61], one study observed positive oral health beliefs (e.g. believing that following recommended oral hygiene is important) in American Indians and Alaskan natives in the US [59].

Oral health behavior also differed between studies. Boggess and colleagues [50] reported that oral hygiene practices significantly varied among ethnicities and races of pregnant women. African American women were more likely than Caucasian and Hispanic women to brush their teeth only once a day or less; and Hispanic women were more likely to use dental floss than Caucasian and African American women. Kiyak et al. [57] reported that Caucasians had a higher risk not to practice positive oral health behaviors than Asians, and a Canadian study observed more often a negative oral health behavior (e.g. never flossing) in Italians compared to those identifying themselves as being Canadian, British, Jewish, or “Other” [53].

## Discussion

To our knowledge, this is the first review that summarizes the research done about OHL and sub-dimensions of OHL (e.g. oral health knowledge, dental service utilization, oral health behaviors and beliefs) of migrants and ethnic/racial minority groups in various host countries. The results of this review show that cultural context and culturally determined beliefs influence the behavior of migrants and ethnic minorities in promoting and maintaining good oral health.

The two studies that aimed to measure OHL in immigrants focused on literacy (reading ability) and observed contrary OHL levels [26, 27]. The reason, probably, is that the sample in one study [26] completed the OHL-assessment in their native language, while the other did not [27]. Similar trends were seen in OHL studies with ethnic minority groups, in which non-Caucasian participants achieved lower literacy scores than their Caucasian counterparts, which was attributed to education and also their proficiency in the language of the host country [28, 30, 32, 33]. Although low education and socioeconomic status has been associated with low HL [62–64], the presently reviewed studies suggest that existing OHL instruments (especially those which only assess functional literacy) may lead to a skewed and incomplete estimation of OHL due to language barriers [65, 66]. Consequently, if the user is not fluent in the language of the host country, OHL-instruments should be provided in the user’s mother tongue. Otherwise, the results would indicate insufficient language skills rather than OHL.

Other important components of OHL, such as culturally influenced oral health attitudes and behaviors, may not be adequately assessed and considered when exploring the overall OHL of immigrant and minority populations. For example, 83.1% of the Brazilian immigrants in the study by Calvasina et al. [26] exhibited adequate numeracy and reading comprehension, but only 29.7% had adequate oral health knowledge. This further supports the idea that despite of adequate functional literacy (as an important component of OHL), there are other relevant factors that play a role in achieving a high OHL.

The results of the studies that explore oral health beliefs, behavior, and service utilization in immigrant and minority groups suggest that the individual cultural background has a significant influence on how migrants and minorities promote and maintain good oral health. In many instances, these cultural influences may attribute to a less than ideal management of oral health. Nonetheless, there have also been instances where populations have exhibited good oral health beliefs [48, 49, 59] and behavior [50, 57], suggesting that the heterogeneous cultural contexts of migrants and ethnic groups can specifically affect one’s health. In fact, research in different populations has observed that oral health beliefs were significantly related to adherence in oral hygiene instructions during periodontal treatment [67] and in preventive dental advice [68]. Health care utilization has also been noted to be lower in immigrants than in native populations, with health beliefs being noted as an understudied, but potentially significant influencing factor [69].

In light of these results, several fields of action arise to improve the OHL in immigrants and minority groups. (Of course, these may count for other health areas and issues as well.) For example, knowledge of risk and severity of oral diseases, benefits of good oral health rather than just avoiding bad oral health, perceived barriers, and measures for improving oral health could be disseminated in a trustful, culturally sensitive way. This may, in turn, increase interest and access to oral health information and services, promote positive oral health behavior and attitudes, support management of oral health, improve patient-doctor interactions, enhance self-efficacy, and thus increase overall OHL. On the health system’s/dental practitioner’s side, continuing training of intercultural competencies in the education of dental students, dentists, and other stakeholders in the provision of oral health care could be provided. In fact, previous research in dental public health has noted that understanding the culture of diverse populations being served is important for the quality of (oral) health care [70] and should be a natural part of the dental curricula [70, 71]. Conveying the importance of these competencies can enhance dental care providers’ interest and will to learn about the specific cultures of their patients. This would be an important basis to increase both the adherence in the dentist-patient-relationship and, as a consequence, the patients’ OHL.

One strength of this systematic review is the inclusion of studies that not only explore OHL explicitly, but also sub-dimensions of OHL that have not been indicated, key worded, or categorized as OHL. This has widened the understanding of OHL or components of OHL, respectively, in migrants and ethnic minority groups, where word recognition tests have been most widely used as a measure of OHL [72]. Another notable aspect of this reviewing process is that the exploration of the research field, the development and conceptualization of the research question, the definition of search terms, and the overall review process itself were conducted by an interdisciplinary team composed of dental practitioners and senior researchers, psychologists, health scientists, and sociologists. This widened the view and allowed for many different aspects and thoughts to be included in the development process.

It should be noted that this review has some potential limitations. Limiting the search to the PubMed database can be seen as one. Not using other databases or grey literature books was decided upon, because most health literacy research related to dentistry would be found in PubMed. We cannot exclude a publication bias that may have resulted from the known fact that significant results are more likely to be published than insignificant results [73]. Although this review is limited to scientific-medical sources, it does likely provide a comprehensive view of the current state of scientific knowledge about OHL.

## Conclusions

Results of this review suggest that cultural context and ethnic affiliation significantly influence migrants’ and ethnic minorities’ behavior in promoting and maintaining good oral health. Although immigrant and minority groups generally showed lower OHL and OHL-related competencies than the native populations, some groups even showed better ones, which underlines the heterogeneity of these different groups, which thus should be handled uniquely. Additionally, our results may suggest that dentists and staff should be aware and open to the possibility that people with a different cultural background have different attitudes, capabilities, and belief systems concerning oral health. Considering these differences should be part of a culturally sensitive approach in medical education and future oral health programs.
